# Long Noncoding RNA NEAT1 as a Potential Candidate Biomarker for Prostate Cancer

**DOI:** 10.3390/life11040320

**Published:** 2021-04-06

**Authors:** Diana Nitusca, Anca Marcu, Alis Dema, Loredana Balacescu, Ovidiu Balacescu, Razvan Bardan, Alin Adrian Cumpanas, Ioan Ovidiu Sirbu, Bogdan Petrut, Edward Seclaman, Catalin Marian

**Affiliations:** 1 Department of Biochemistry and Pharmacology, “Victor Babeş” University of Medicine and Pharmacy, Pta Eftimie Murgu Nr. 2, 300041 Timişoara, Romania; nitusca.diana@umft.ro (D.N.); marcu.anca@umft.ro (A.M.); ovidiu.sirbu@umft.ro (I.O.S.); eseclaman@umft.ro (E.S.); 2 Department of Pathology, “Victor Babeş” University of Medicine and Pharmacy, Pta Eftimie Murgu Nr. 2, 300041 Timişoara, Romania; dema.alis@umft.ro; 3 Department of Genetics, Genomics and Experimental Pathology, The Oncology Institute “Prof. Dr. Ion Chiricuta”, 400015 Cluj-Napoca, Romania; lbalacescu@iocn.ro (L.B.); ovidiubalacescu@iocn.ro (O.B.); 4 Department of Urology, “Victor Babeş” University of Medicine and Pharmacy, Pta Eftimie Murgu Nr. 2, 300041 Timişoara, Romania; razvan.bardan@umft.ro (R.B.); cumpanas.alin@umft.ro (A.A.C.); 5 Urology Clinic, Timisoara Emergency County Hospital, 300723 Timisoara, Romania; 6 Department of Urology, The Oncology Institute “Prof. Dr. Ion Chiricuta”, 400015 Cluj-Napoca, Romania; bogdan.petrut@umfcluj.ro

**Keywords:** prostate cancer, long noncoding RNA, NEAT1, laser capture microdissection

## Abstract

*Background:* Prostate cancer (PCa) remains one of the leading causes of cancer-related mortality in men worldwide, mainly due to unsatisfactory diagnostic methods used at present, which lead to overdiagnosis, unnecessary biopsies and treatment, or misdiagnosis in early asymptomatic stages. New diagnostic biomarkers are needed for a correct and early diagnosis. Long noncoding RNAs (lncRNAs) have been broadly studied for their involvement in PCa biology, as well as for their potential role as diagnostic biomarkers. *Methods:* We conducted lncRNA profiling in plasma and microdissected formalin-fixed paraffin-embedded (FFPE) tissues of PCa patients and attempted validation for commonly dysregulated individual lncRNAs. *Results:* Plasma profiling revealed eight dysregulated lncRNAs, while microarray analysis revealed 717 significantly dysregulated lncRNAs, out of which only nuclear-enriched abundant transcript 1 (NEAT1) was commonly upregulated in plasma samples and FFPE tissues. NEAT1’s individual validation revealed statistically significant upregulation (FC = 2.101, *p* = 0.009). Receiver operating characteristic (ROC) analysis showed an area under the curve (AUC) value of 0.7298 for NEAT1 (95% CI = 0.5812–0.8785), suggesting a relatively high diagnostic value, thus having a potential biomarker role for this malignancy. *Conclusions:* We present herein data suggesting that NEAT1 could serve as a diagnostic biomarker for PCa. Additional studies of larger cohorts are needed to confirm our findings, as well as the oncogenic mechanism of NEAT1 in the development of PCa.

## 1. Introduction

Prostate cancer (PCa) currently represents one of the leading causes of cancer-related mortality among men worldwide [1], with an incidence rate of almost 60% over the age of 65 years [2]. Notwithstanding the great effort of the research field and the important contributions that modern medicine implemented over the past decades, the progress in reducing PCa mortality remains disputable to a certain extent [3]. 

The American Cancer Society predicts a total number of about 191,930 estimated new cases of PCa for 2020, with an increase of over 17,000 cases compared to the estimations of 2019 [4]. In addition, the mortality rate due to PCa is also expected to increase, with 33,330 estimated deaths in 2020 alone in the US, which surpasses the number of deaths from previous recent years. Worldwide statistics of PCa revealed that 3.8% of all deaths caused by cancer in men were due to PCa, with 358,989 deaths and 1,276,106 new cases in 2018 [5,6]. 

The high mortality rate could be at least partly explained due to the asymptomatic nature of PCa in the early stages, which leads to late diagnosis in most cases. Currently, several biomarkers are considered useful for PCa diagnosis and prognosis. However, only a few of them, such as prostate-specific antigen (PSA)-based, were Food and Drug Administration (FDA)-approved and used as PCa biomarkers in clinical use [7]. Nevertheless, even if PSA-based tests are useful for PCa diagnosis, due to their highly organ-specificity, PSA is not a cancer-specific biomarker, being also increased in benign prostatic hyperplasia (BPH) inflammation, body weight, lifestyle factors, or physical manipulation [8,9]. Consequently, the use of prostate-specific antigen (PSA) analysis, due to its low specificity for PCa, would adversely impact overdiagnosis, overtreatment, and unnecessary biopsies [10]. 

Therefore, the limitations of the diagnostic strategies that are currently used in matters of PCa require the identification of new approaches for novel diagnostic and prognostic biomarkers that could aid in the fine-tuning of conventional serum biomarkers [11]. The noninvasive liquid biopsy technique attempts to overcome the disadvantages and impediments of the current approaches, both for patient comfort and clinical utility [12]. DNA-, RNA-, and protein-based biomarkers represent promising candidates for future large-scale screenings, from which some already showed clinical relevance [13]. It is the case for prostate cancer antigen 3 (PCA3), a type of lncRNA, which outperformed PSA testing in matters of specificity [14]. 

LncRNAs are transcripts over 200 nucleotides in length, which are generally not translated into proteins [15]. They have shown to play different roles in physiology, such as development and differentiation, acting as transcription regulators. They function as enhancer RNAs, decoys, signals, guides in order to modulate transcription via chromatin remodeling, and sequestering regulatory factors [16,17]. Like other noncoding RNA species, lncRNAs are dysregulated in a vast number of medical conditions (i.e., psoriasis, Alzheimer’s disease) and in cancer (breast cancer, colon cancer, PCa, etc.) [18,19,20,21,22]. They possess excellent features such as having specific prostate tissue expression and being localized to certain subcellular regions [23,24]. Moreover, various lncRNAs have shown to have a differential expression level compared to healthy controls (HCs), suggesting a likely diagnostic biomarker potential that could represent a novel, more optimized, and noninvasive approach for the diagnosis of PCa [25].

Herein, we performed lncRNA profiling in plasma and FFPE samples of PCa patients to analyze the lncRNA relative expression. We attempted to validate individual lncRNA NEAT1 as a diagnostic biomarker for PCa. Additionally, by using laser capture microdissection (LCM) as a valuable technology for limiting the heterogeneity from FFPE samples, we were capable of isolating only the desired areas of interest from a very diverse and heterogeneous tissue specimen.

## 2. Materials and Methods

### 2.1. Study Design 

**Figure 1:**
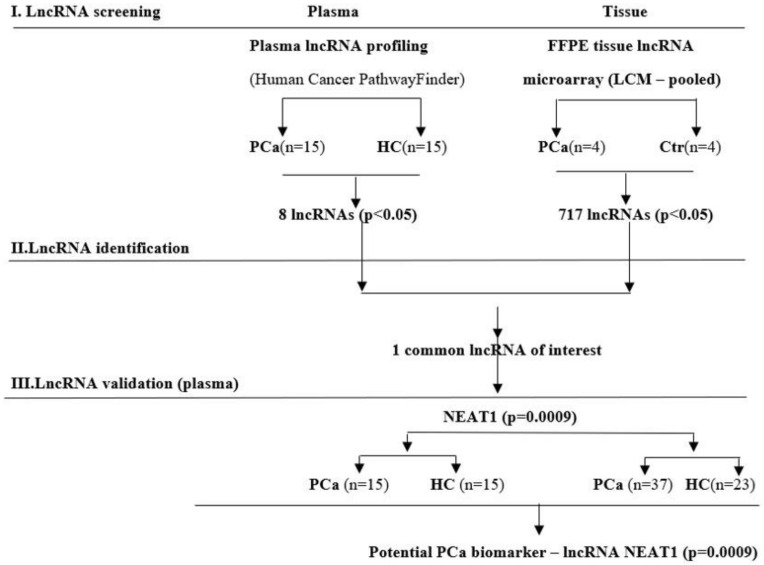
Flow-diagram of the study design used to screen, identify, and validate new lncRNA as prostate cancer (PCa) specific biomarkers.

Our study design included a stage of lncRNA screening using both plasma and tissue samples, followed by two identification and validation stages on plasma samples. A flow-diagram representation of our study is shown in Figure 1.

Firstly, we conducted a lncRNA screening in plasma of 15 PCa patients and 15 healthy controls (HCs) and in 8 laser capture microdissected (LCM) FFPE tissues of PCa and adjacent normal tissues. Only NEAT1 was the commonly and significantly (*p* < 0.05) upregulated lncRNA among groups. It was further validated in an individual assay, consisting of the previous group of 15 patients with PCa and 15 HC plus an additional group of 37 PCa patients and 23 HC. 

Individual validation of NEAT1 was performed in two different groups of patients and controls, from two participating institutions: “Victor Babeş” University of Medicine and Pharmacy Timisoara (designated TM) and The Oncology Institute “Prof. Dr. Ion Chiricuta” (designated CJ).

### 2.2. Patients’ Characteristics and Plasma Samples

The biological samples (blood and tissues) used for screening stages were collected from the Urology Clinic of the Clinical Emergency County Hospital in Timisoara, Romania, while the plasma samples used for validation were provided by the Oncology Institute “Prof Dr. Ion Chiricuta” Cluj-Napoca. Patients admitted for the screening stage had undergone transrectal biopsies for histopathological diagnosis of PCa. Control samples were collected from healthy subjects, with no prostate disease, from the same hospital. All HCs had normal PSA levels (<4 ng/mL), verified by chemiluminescent microparticle immunoassay (Abbott Diagnostics, Chicago, IL, USA). 

All subjects included in this study provided informed consent for the use of their biological samples. The study was approved by the Ethics Committee of the participating institutions, in accordance with the Declaration of Helsinki, and venous blood was collected in EDTA-containing tubes, as previously described [26]. Patients’ characteristics are briefly summarized in Table 1.

### 2.3. Plasma lncRNA Screening

According to the manufacturer’s instructions, total RNA was isolated from plasma using the miRNeasy Serum/Plasma Advanced Kit (Qiagen, Hilden, Germany). RNA concentration and quality were verified using a NanoDrop ND-1000 spectrophotometer (Thermo Fisher Scientific, Waltham, MA, USA). Reverse-transcription was performed using RT^2^-PreAMP cDNA Synthesis (Qiagen, Germany) to obtain the cDNA sequence, with a starting quantity of 60 ng RNA, according to the manufacturer’s indications. cDNA was preamplified using specific primers, with the RT2 lncRNA PreAMP Primer Mix for Human Cancer PathwayFinder kit (Qiagen, Germany). Real-time PCR analysis for multiple lncRNAs was performed on a 7900 HT Real-Time PCR System (Thermo Fisher Scientific, USA), using RT2 lncRNA PCR Array Human Cancer PathwayFinder (Qiagen, Germany) combined with RT^2^ SYBR Green qPCR Mastermix (Qiagen, Germany), for lncRNA profiling, following the manufacturer’s protocol. 

### 2.4. Plasma lncRNA Validation

Differentially expressed lncRNAs were further validated using TaqMan® Fast Advanced Master Mix (Thermo Fisher Scientific, USA) and specific primers. RNA was extracted from plasma using miRNeasy Serum/Plasma Advanced Kit (Qiagen, Germany), according to the manufacturer’s instructions. Reverse-transcription was performed using SuperScript™ VILO™ cDNA Synthesis Kit (Thermo Fisher Scientific, USA). cDNA was subsequently used as a template in a Veriti 96-Well Thermal Cycler (Applied Biosystems, Foster, CA, USA) compatible with all kits used, following the manufacturer’s suggestions. All samples were performed in triplicate. 

### 2.5. LncRNA Analysis in FFPE Tissues

Eleven-year-old FFPE tissue samples of PCa from the Department of Pathology, University of Medicine and Pharmacy “Victor Babes” Timisoara, were sectioned (10 µm in size), mounted on MMI RNAse-free slides (MMI, Zurich, Switzerland), and microdissected using LCM technology, as previously described [26,27].

Total RNA was extracted from the tissue samples using miRNAeasy FFPE kit, with a melting protocol (Qiagen, Germany), according to the manufacturer’s indications. Eight pooled tumor and normal adjacent samples, with enough RNA amount and good integrity evaluated by NanoDrop ND-1000 (Thermo Fischer Scientific, USA) and Agilent 2100 Bioanalyzer (Agilent Technologies, Santa Clara, CA, USA), were subjected to microarray analysis.

Each RNA sample (100 ng) was amplified and labeled with Cy3 using the Low Input Quick Amp Labeling Kit (Agilent Technologies). The Cy3-labeled cRNA probes were hybridized on SurePrint G3 Human GE v3 8 × 60 K arrays (Agilent Technologies) for 17 h at 65 °C. After washing, arrays were scanned with Agilent G2505C Microarray Scanner at 3 µm resolution and image files were processed with Agilent Feature Extraction software v. 11.5.1.1 (Agilent Technologies, Palo Alto, CA, USA).

### 2.6. Statistical Analysis

Results from the plasma lncRNA profiling step were analyzed using the statistical analysis platform GeneGlobe Data Analysis Center (Qiagen, Germany). Raw Cq values were preprocessed setting 37 as cutoff value and expression in at least 80% of samples. Ct values were normalized via an automatic selection of housekeeping genes. *ACTB* was used as endogenous control in plasma analysis for both profiling and validation. Relative quantities were log-transformed and compared (PCa vs. HC) among groups. The p-values were calculated using the Student’s t-test of the replicate 2^ (-Delta CT) values. Individual lncRNAs were analyzed by the ∆∆Ct method for each gene in the PCa and HC groups.

Microarray data analysis was performed in R/Bioconductor. Raw median signals were filtered, background corrected and quantile normalized between arrays. The median value of all probes for each transcript was calculated. The differential expression was tested with limma package using the following criteria: absolute fold change >1.5 and *p* < 0.05. 

## 3. Results

All subjects’ clinical data are presented in Table 1. A certified pathologist analyzed the archived FFPE tissue samples to confirm PCa diagnosis, but no clinical and demographic characteristics are available for the eleven-year-old archived samples.

The results from the profiling step showed a total number of eight differentially expressed lncRNAs in plasma of PCa patients compared to healthy subjects, from which three were upregulated and five were downregulated, as shown in Table 2.

FFPE tissue microarray analysis revealed 717 lncRNAs that were markedly dysregulated in PCa samples versus controls (data not shown).

The comparison between FFPE tissues and plasma samples showed only one commonly upregulated lncRNA, NEAT1 (*p* < 0.05).

**Figure 2:**
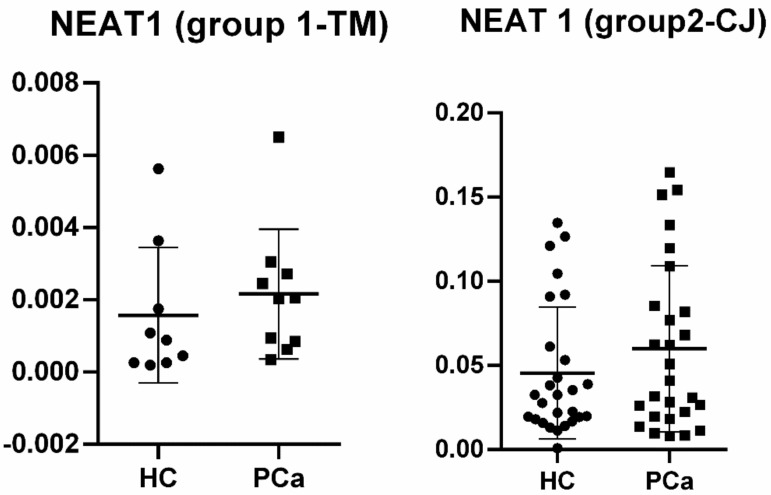
Relative quantities for NEAT1 in plasma of prostate cancer (PCa) patients vs. healthy controls (HCs) among groups.

In the next step, NEAT1 was individually validated in plasma and tissue samples from both groups, as its expression was commonly and significantly upregulated in PCa subjects when compared to HC. In plasma samples from Group 1 (TM), although NEAT1 was upregulated (FC = 1.836), it did not reach statistical significance (*p* = 0.351). In contrast, in Group 2 (CJ), where the sample size was larger, NEAT1 was significantly upregulated (FC = 2.101, *p* = 0.009). Figure 2 shows the relative quantities for both groups. 

**Figure 3:**
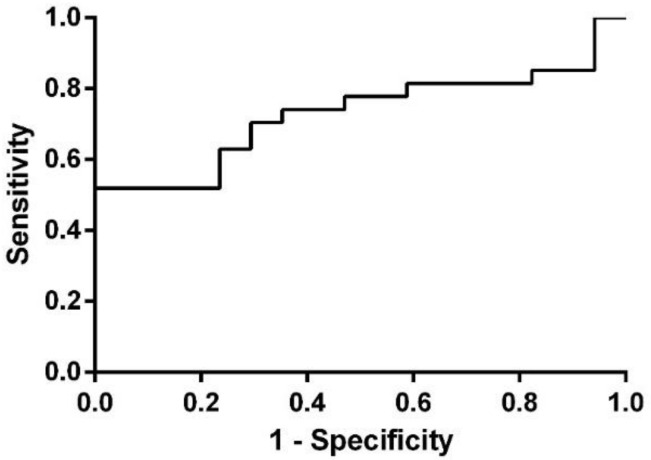
ROC analysis for NEAT1 in Group 2 (CJ).

ROC analysis for NEAT1 in Group 2 (CJ) revealed an area under the curve (AUC) of 0.7298 (95%CI = 0.5812–0.8785), suggesting, therefore, the biomarker potential for this type of lncRNA (Figure 3).

## 4. Discussion

Our study aimed to investigate differentially expressed lncRNA species in PCa patients’ plasma and LCM FFPE tissue samples, compared to healthy controls (HC), to determine lncRNAs as potential diagnostic biomarkers for PCa. The common lncRNA that was found to be dysregulated in both groups was nuclear-enriched abundant transcript 1 (NEAT1), and therefore, we conducted an individual analysis in order to validate this type of lncRNA as a biomarker for prostate malignancy. In Group 1 (TM), individual validation did not reach statistical significance (*p* = 0.351), most probably because of the low sample size, although it was upregulated with an almost two-fold increase in PCa samples when compared to HC. In Group 2 (CJ), NEAT1 revealed to be also upregulated (FC = 2.101), and this time, the result presented statistical significance (*p* = 0.009) and an AUC value of 0.7298 (95%CI = 0.5812–0.8785).

Our study went in the same direction as what was previously published in the literature. A report conducted by Li et al. (2018) proved the oncogenic role (and consequent overexpression) of NEAT1 and its functionality dependence on certain transcription factors [28]. To date, it is known that NEAT1 is an essential component for the structure of paraspeckles (nuclear domains that have a role in nuclear retaining of mRNA). This abundant 4kb lncRNA increased the numbers of paraspeckles when overexpressed and eradicated them when depleted by RNAi, respectively [23]. Besides this architectural role, NEAT1 showed to be involved in various processes related to cancer, such as invasion, migration, proliferation, DNA damage, etc. [29], but the concrete tumorigenesis mechanism of NEAT1 remains unclear.

However, a new transcription regulation mechanism has been proposed, proving that the oncogenic role of NEAT1 is highly dependent on the transcriptional regulatory circuit NEAT1-CDC5L-AGRN. Cell division cycle 5-like protein (CDC5L) is essential for mitotic progression, and its target gene, AGRN, seems to be modulated by NEAT1, yielding this whole pathway critical for tumor growth [28].

Another oncogenic pathway for NEAT1 was proposed by Xiong et al. (2018), who showed that NEAT1 promotes PCa cell growth via the SRC3/IGF1R/AKT pathway. In this manner, NEAT1 interacts with steroid receptor co-activator3 (SRC3), therefore upregulating the phosphorylation of AKT and promoting PCa cell growth via IGF1R/AKT pathway. NEAT1 was consequently found to be overexpressed in PCa samples, together with SRC3 and IGF1R [30].

Yet, another study confirms the oncogenic potential of NEAT1, proving that it is the most upregulated lncRNA in PCa samples. In addition, NEAT1 showed to be recruited at the sites of PCa genes where it contributes, on an epigenetic level, to the promotion of tumorigenesis. The same study demonstrates that NEAT1 is a potential target for estrogen receptor alpha (ERα), suggesting that ERα could function as an alternate signaling pathway that can help refractory PCa bypass the classical androgen/androgen receptor (AR) axis [31].

In addition, responding to the emergent need of identifying mechanisms of lncRNAs, recent reports suggest that NEAT1 acts as a sponge for miR-98-5p to upregulate the oncogene HMGA2, proving that another novel regulatory pathway (NEAT1-miR-98-5p-HMGA2) could be crucial for PCa development [32].

Moreover, a pan-cancer analysis showed the same tendency of NEAT1 to be overexpressed in various types of cancer, besides PCa, such as stomach adenocarcinoma, hepatocellular liver carcinoma, kidney papillary cell carcinoma, and kidney clear cell carcinoma, although some contradictory evidence exists regarding its tumor suppressor role in promyelocytic leukemia [33,34].

To our knowledge, this is the first study that investigated the differential expression of NEAT1 in PCa FFPE tissues, using the LCM technique. Using this tool for isolating desired cell populations, we increased the biomarker specificity by limiting the sample heterogeneity, thus minimizing the risk of introducing noncancer cells that could interfere with the data obtained for the relative quantification of NEAT1 in FFPE PCa tissue samples. However, our study comprised a small sample size, which represents its main limitation, together with the lack of multiple comparison correction.

Taken together, these findings corroborate with previous reports stating that NEAT1 could be used as a biomarker for PCa diagnosis and should be perceived in the large context of biomarker discovery using novel and modern medical approaches. Undoubtedly, future studies comprising larger cohorts are compulsory for better understanding the roles and mechanisms of NEAT1 as an oncogene for PCa development, as well as the reliability of its overexpression in PCa samples (plasma, tissues, etc.) when compared to healthy subjects.

## 5. Conclusions

Our findings demonstrate that NEAT1 is significantly upregulated in PCa samples compared to HC, suggesting an oncogenic role for this particular type of lncRNA. Analyzed in an individual validation study, NEAT1 showed to have a relatively high diagnostic value and, therefore, could represent a promising and novel biomarker for PCa detection. However, these data need to be confirmed with the aid of additional studies encompassing larger cohort sizes that could ultimately lead to discovering the comprehensive oncogenic mechanism of NEAT1 regarding PCa biology.

## Figures and Tables

**Table 1:** Clinical and demographic characteristics of the subjects included in the study.

Characteristics	Training Lot		Validation Lot	
	Patients (n = 15)	Controls (n = 15)	Patients (n = 37)	Controls (n = 23)
**Age (±SD)**	67.2 (±4.18)	51.3 (±8.27)	65 (±6.96)	58.2 (±9.51)
**PSA n (%)**				
**<4 ng/mL**	0 (0.00)	15 (100.00)	2 (5.41%)	-
**4–10 ng/mL**	5 (33.33) *	0 (0.00)	20 (54.05 %)	-
**≥10 ng/mL**	9 (60.00) *	0 (0.00)	15 (40.54%)	-
**Gleason score n (%)**				
**5–6**	3 (20.00)		11 (29.7%)	
**7**	9 (60.00)		22 (59.4%)	
**8–10**	3 (20.00)		4 (10.9%)	

* Does not add up to 100% due to missing values.

**Table 2:** Differentially expressed lncRNAs between PCa subjects and healthy controls (HCs).

LncRNA	Fold Change (FC)	*p*-Value
MIR7-3HG	130.67	0.011
NEAT1	66.94	0.029
RMRP	2.55	0.034
CAHM	−885.36	0.0009
CCAT1	−5.63	0.038
CCAT2	−88.02	0.0007
MRPL23-AS1	−7.40	0.017
XIST	−93.62	0.031
